# Latent profile analysis and influencing factors of quality of life in pregnant women with gestational diabetes mellitus

**DOI:** 10.1186/s12884-023-06079-2

**Published:** 2023-11-11

**Authors:** Xin-yi Zhou, Yan-feng Wang, Jie-mei Yang, Li-yuan Yang, Wei-jia Zhao, Yan-ling Chen, Qiao-hong Yang

**Affiliations:** 1 School of Nursing, Jinan University, Guangzhou, Guangdong 510632 China; 2 Department of Obstetrics, Zhuhai Maternal and Child Health Hospital, Zhuhai, Guangdong 519001 China; 3 Guangzhou Women and Children’s Medical Center, Guangzhou, Guangdong 510620 China; 4 School of Health, Dongguan Vocational and Technical College, Dongguan, Guangdong 523808 China

**Keywords:** Gestational Diabetes Mellitus, Pregnant woman, Quality of life, Latent profile analysis

## Abstract

**Purpose:**

Gestational diabetes mellitus (GDM) negatively affects the quality of life of pregnant women and is influenced by several factors. Research to date treats pregnant women with gestational diabetes as a homogeneous group based on their quality of life. We attempted to identify subgroups based on self-reported quality of life and explored variables associated with subgroups.

**Methods:**

From September 1, 2020 to November 29, 2020, pregnant women with GDM from two hospitals in Guangdong Province were selected as subjects by convenience sampling method. Medical records provided sociodemographic data, duration of GDM, pregnancy status, and family history of diabetes. Participants completed validated questionnaires for quality of life, anxiety and depression. Latent profile analysis was used to identify profiles of quality of life in pregnant women with GDM, and then a mixed regression method was used to analyze the influencing factors of different profiles.

**Results:**

A total of 279 valid questionnaires were collected. The results of the latent profile analysis showed that the quality of life of pregnant women with GDM could be divided into two profiles: C1 “high worry-high support” group (75.6%) and C2 “low worry-low support” group (24.4%). Daily exercise duration and depression degree are negative influencing factors, making it easier to enter the C1 group (p < 0.05). Disease duration and family history of diabetes are positive influencing factors, making it easier to enter the C2 group (p < 0.05).

**Conclusion:**

The quality of life of pregnant women with GDM had obvious classification characteristics. Pregnant women with exercise habits and depression are more likely to enter the “high worry-high support” group, and health care providers should guide their exercise according to exercise guidelines during pregnancy and strengthen psychological intervention. Pregnant women with a family history of diabetes and a longer duration of the disease are more likely to fall into the “low worry-low support” group. Healthcare providers can strengthen health education for them and improve their disease self-management abilities.

## Introduction

Gestational diabetes mellitus (GDM) is defined as glucose intolerance of any degree first identified during pregnancy and is typically diagnosed during the second trimester at 24–28 weeks of gestation [1]. GDM is recognized as one of the fastest-growing forms of diabetes due to increasing rates of obesity and maternal age worldwide [2]. The latest data from the International Diabetes Federation show that in 2021, 1 in 6 live births worldwide were affected by maternal hyperglycemia during pregnancy—80.3% of which were due to GDM [3].

GDM poses many serious risks and adverse outcomes for pregnant women and their infants. For pregnant women, the prevalence of complications such as pre-eclampsia, hypertension, hyper amniotic fluid, premature rupture of membranes, cesarean section, and depression increases [4, 5]. Long-term, the risk of type 2 diabetes mellitus in pregnant women with GDM increases by 7 times, that of cardiovascular disease increases by 4 times, and that of metabolic syndrome, malignant tumors, kidney disease, and eye disease also increase [6, 7]. When these women get pregnant again, the recurrence rate of GDM is as high as 33–69% [8]. Infants born to pregnant women with GDM are at increased risk of large-for-gestational-age, intrauterine growth restriction, hypoglycemia, hyperbilirubinemia, and neonatal respiratory distress syndrome, as well as extended length of stay in the neonatal intensive care unit [9]. In addition, these babies are also risky for long-term bad health results, including impaired neurodevelopment, difficulty maintaining a healthy body mass index (BMI), and increased risk of type 2 diabetes, obesity, cardiovascular disease, and psychiatric disorders [6].

The above-mentioned risks and adverse outcomes force pregnant women with GDM not only bear the physical and psychological distress of the disease but also concern for the safety and prognosis of the baby. In addition, the behavioral restrictions as a result of the disease have a certain impact on the social activities and work lives of these pregnant women, and the cost of disease treatment also increases their family economic burden to varying degrees [10]. For healthcare providers, disease treatment increases the frequency of medical resource consumption among pregnant women with GDM, and they need to provide more support to meet the health service needs of pregnant women with GDM [10]. All of these outcomes seriously affect the quality of life (QOL) of pregnant women with GDM [4–10].

QOL as an individual’s perception of their position in life in the context of the culture and value systems in which they live and in relation to their goals, expectations, standards and concerns [11]. Health-related QOL represents a measure of physical and social activity as well as mental health, and is considered an important health indicator [8]. Nowadays, the evaluation and recording of health-centered QOL through QOL questionnaires have become key in medical and nursing interventions [12]. Scholars have developed and used the Quality of Life Scale for Women with Gestational Diabetes mellitus (GDMQ) to measure the QOL of pregnant women with GDM, and extensively explored its influencing factors based on the comprehensive score [12, 13]. Studies have shown that the overall QOL of pregnant women with GDM is usually at a moderately low level, and about a quarter of pregnant women have poor QOL. Individual-specific variables such as demographic variables, pregnancy- and disease-related variables, anxiety, and depression are important factors affecting the QOL of pregnant women with GDM [11, 13, 14].

Although previous studies have confirmed that demographic variables, pregnancy and disease-related variables, and psychological variables have important effects on the QOL of pregnant women with GDM, these studies mostly adopted a variable-centered approach, that is, to judge the level of QOL according to the total score of the scale. Previous studies have found large differences between the maximum and minimum GDMQ scores in pregnant women with GDM, which suggests that there may be heterogeneous subgroups in the QOL of women with GDM [13]. However, the variable-centered approach treats all pregnant women as a homogeneous group and cannot discern heterogeneous differences [15]. To help clinics formulate more targeted interventions, latent profile analysis (LPA) is a more suitable method for evaluating the QOL of pregnant women with GDM. LPA is a people-oriented method that can group individuals with similar reactions into the same category, judge potential characteristics according to the answers of different profiles on the scale items, and understand the proportion of different profiles compared to the whole [16]. By focusing on individual heterogeneity, LPA is helpful to understand the characteristics and influencing factors of different potential profile populations. Several studies have used LPA to assess the QOL of study subjects, and they concluded that the use of LPA can help to better tailor interventions, especially those for members of specific subgroups [15, 17, 18].

To our knowledge, no studies have used LPA to analyze QOL in pregnant women with GDM. Therefore, this study aims to use LPA to classify the QOL patterns of pregnant women with GDM, and analyze the differences in sociodemographic characteristics, pregnancy status, family history of diabetes, anxiety, and depression factors of each profile, in order to provide guidance for improving the quality of life of pregnant women with gestational diabetes mellitus.

## Methods

### Design and participants

This study has been approved by the Ethics Committee of Guangzhou Women and Children’s Medical Center (Ref. 046A01). The convenience sampling method was used to select pregnant women with GDM who were admitted to two tertiary hospitals in Guangdong Province. Inclusion criteria were as follows: (1) aged ≥ 18 years; (2) diagnosed with GDM according to the criteria published by the International Diabetes Association and the Pregnancy Research Group [3]; (3) pregnant women whose GDM duration does not exceed one month. Exclusion criteria were as follows: (1) pregnant women with pre-existing diabetes; (2) presence of other high-risk pregnancy conditions and psychopathology; (3) participation in other intervention research. A study by Yi et al. [19] showed that robust statistical results can be obtained when the average sample size of each profile reaches 50. As the maximum number of profiles to be explored in this study is 5, the required sample size is at least 250 pregnant women. A total of 279 women with GDM were included in this study for analysis.

### Measures

Data was captured in two ways. First, from the subjects’ medical records. Second, it was captured from a set of self-administered questionnaires completed by the subjects. Medical records provided sociodemographic data (age, working status, educational level, place of residence, and per-capita monthly household income, BMI, daily exercise duration, and daily sleep duration), duration of GDM, pregnancy status(pregnancy stage, pregnancy status, number of fetal fetuses, pregnancy methods), and family history of diabetes.

### Quality of life scale for women with gestational Diabetes Mellitus (GDMQ)

The Chinese version of GDMQ was used to measure QOL in pregnant women. GDMQ was compiled by Simbar et al. [12] in 2019 and translated into Chinese by Yang et al. [20] in 2021. The Chinese version of GDMQ includes 32 items and the following five dimensions: concerns about pregnancy risk factors (10 items), perceived limitations (7 items), gestational diabetes complications (4 items), medication and treatment (6 items), and support (5 items), these are measured on a 5-point Likert scale from 1 (strongly disagree) to 5 (strongly agree). The item “I adjust the insulin dose according to the blood sugar condition” and all items in the support dimension adopt the forward scoring method, and the remaining items adopt the reverse scoring method. The total score of the 32 items is considered the original score, and the standard score is obtained according to the following formula: (original score/160)*100. Thus, the total standard score is 20–100, where higher scores represent better QOL. In this study, Cronbach’s α coefficient was 0.914. This scale is suitable for investigating the quality of life of pregnant women with gestational diabetes mellitus [20].

### Self-rating anxiety scale (SAS)

The Chinese version of SAS was used to assess the degree of anxiety in pregnant women. SAS was compiled by Zung et al. [21] in 1971 and translated into Chinese by Wang et al. [21] in 1984. SAS includes 10 items, these are measured on a 5-point Likert scale from 1 (none) to 4 (always). The total score of 20 items is considered the original score, and the integral part of the original score multiplied by 1.25 is considered the standard score. The evaluation result was determined by standard score: scores of < 50 indicated no anxiety; scores of ≥ 50 and < 70 indicated mild anxiety; scores of ≥ 70 and < 85 indicated moderate anxiety; and scores of ≥ 85 indicated severe anxiety. In this study, Cronbach’s α for the Chinese version of SAS was 0.82, which was suitable for screening the perinatal anxiety of pregnant women [22].

### Edinburgh Postpartum Depression Scale (EPDS)

The Chinese version of the EPDS was used to assess the degree of depression in pregnant women. The EPDS was compiled by Cox et al. [23] in 1987 and translated into Chinese by Lee et al. [24] in 1988. EPDS includes 10 items, these are measured on a 4-level scoring method, with scores ranging from 1 to 4. The total score is 0–30, where a score of ≥ 13 indicates depression, and higher scores represent more severe depression. In this study, Cronbach’s α of the Chinese version of EPDS was 0.76, indicating that it can be used to screen women with possible depression during pregnancy [25].

#### Data collection

From September 1, 2020 to November 29, 2020, two investigators conducted on-the-spot investigations on patients in obstetrics departments of two hospitals in Guangdong Province. The investigators were uniformly trained according to the requirements of this study. First, investigators invited patients who met the inclusion and exclusion criteria to participate in the study. Then, participants gave consent and signed the informed consent form. Finally, investigators distributed questionnaires on the spot. If participants could not read or write, the questionnaire was completed with the help of the investigator, who read out each question and recorded the responses of the participants. All questionnaires were collected and checked by two investigators onsite.

### Statistical analysis

Descriptive analysis was performed using SPSS 26.0 (IBM Corporation, Armonk, NY, USA). LPA was performed using Mplus8.0 (Muthen & Muthen, Los Angeles, CA, USA). A three-step approach was adopted to perform LPA. First, LPA was performed with the mean of the scores of the five dimensions in GDMQ as the explicit variable. The best model was selected according to the model evaluation index and clinical significance. Akaike information criterion (AIC), Bayesian information criterion (BIC), and adjusted Bayesian information criterion are indicators used to evaluate the fitting degree of the model, where the smaller the above-three values, the better the fitting degree of the model [26]. The entropy value is an indicator for evaluating the classification accuracy of the model; it ranges from 0 to 1, where values closer to 1 indicate a higher accuracy [26]. In addition, the difference in model fit was verified by the Lo–Mendell–Reuben-corrected likelihood ratio (LMR) and the Bootstrap-based likelihood ratio (BLRT), where p < 0.05 indicated that the kth category model was better than the k–1th category model [26]. Then, suitable class memberships are obtained according to the posterior distribution from the previous step [27]. Chi-square and nonparametric tests were used to identify variables with differences between profiles. Finally, a regression mixture model was constructed and regression analysis was performed using the R3STEP command in Mplus to determine the influencing factors for each profile.

## Results

A total of 288 questionnaires were distributed in this study. Among them, 9 questionnaires had incomplete responses and were regarded as invalid questionnaires and were eliminated. Complete responses were considered valid questionnaires, with a total of 279 valid questionnaires. The effective response rate of the questionnaire was 96.86%. The average age of the 279 pregnant women was 32.05 ± 4.52 years old, and they were first diagnosed with GDM at 24–28 weeks of pregnancy. The other general information is shown in Table 1.

**Table 1:** Participant characteristics (n = 279)

Name of variable	Categories	Total Sample N (%)	Profiles		*χ* ^*2*^	*p-*value
			Class1 (n = 211)	Class2 (n = 68)		
Age	≤ 30	77(27.60)	57(27.01)	20(29.41)	0.148	0.701
	>30	202(72.40)	154(72.99)	48(70.59)		
Working status	Working	188(67.38)	142(67.30)	46(67.65)	0.003	0.957
	Not working	91(32.62)	69(32.70)	22(32.35)		
Education level	Primary school and below	7(2.51)	5(2.37)	2(2.94)	1.017	0.797
	Middle School and Vocational High School	70(25.09)	55(26.07)	15(22.06)		
	College and undergraduate	187(67.03)	141(66.82)	46(67.65)		
	Postgraduate and above	15(5.38)	10(4.74)	5(7.35)		
Place of residence	Village	44(15.77)	35(16.59)	9(13.24)	1.924	0.382
	Town	83(29.75)	66(31.28)	17(25.00)		
	City	152(54.48)	110(52.13)	42(61.76)		
Per-capita monthly household income (RMB, yuan)	<5000	53(19.00)	41(19.43)	12(17.65)	2.798	0.424
	5000–10,000	132(47.31)	104(49.29)	28(41.18)		
	10,000–20,000	65(23.30)	47(22.27)	18(26.47)		
	>20,000	29(10.39)	19(9.00)	10(14.71)		
BMI before pregnancy	<18.5	37(13.26)	28(13.27)	9(13.24)	1.274	0.735
	18.5–24.9	193(69.18)	143(67.77)	50(73.53)		
	25–29.9	44(15.77)	36(17.06)	8(11.76)		
	≥ 30	5(1.79)	4(1.90)	1(1.47)		
Daily exercise duration (h)	None	45(16.13)	16(7.58)	29(42.65)	49.576	0.000
	<1	129(46.24)	102(48.34)	27(39.71)		
	≥ 1	105(37.63)	93(44.08)	12(17.65)		
Daily sleep duration (h)	<6	9(3.23)	7(3.32)	2(2.94)	0.537	1.000
	6–8	168(60.22)	126(59.72)	42(61.76)		
	8–10	99(35.480	75(35.55)	24(35.29)		
	>10	3(1.08)	3(1.42)	0(0.00)		
Disease duration (week)	1	67(24.01)	48(22.75)	19(27.94)	12.511	0.006
	2	46(16.49)	44(20.85)	2(2.94)		
	3	50(17.92)	34(16.11)	16(23.53)		
	4	116(41.58)	85(40.28)	31(45.59)		
Pregnancy stage	Middle pregnancy	132(47.31)	102(48.34)	30(44.12)	0.368	0.544
	Late pregnancy	147(52.69)	109(51.66)	38(55.88)		
Pregnancy status	Primigravid	131(46.95)	94(44.55)	37(54.41)	0.202	0.654
	Multigravid	148(53.05)	117(55.45)	31(45.59)		
Number of fetuses	1	267(95.70)	201(95.26)	66(97.06)	1.080	0.368
	2	9(3.23)	8(3.79)	1(1.47)		
	≥ 3	3(1.08)	2(0.95)	1(1.47)		
Method of conception	Artificial insemination	29(10.39)	23(10.90)	6(8.82)	0.238	0.626
	Natural conception	250(89.61)	188(89.10)	62(91.18)		
Family history of diabetes	Yes	111(39.78)	60(28.44)	51(75.00)	46.544	0.000
	No	168(60.22)	151(71.56)	17(25.00)		
Anxiety		40(35, 45)	40(36, 45)	40(35, 45)	8.556^a^	0.000
Depression		6(3, 10)	8(5, 11)	2(1, 5)	0.544^a^	0.586

^a^ Mann–Whitney U Test

The participants average total GDMQ score was 58.87 ± 10.38, and the five dimensions were scored as follows: concerns about high-risk pregnancy (17.08 ± 6.12), perceived constraints (11.58 ± 3.94), disease complications (7.69 ± 1.98), medication and treatment (11.67 ± 2.13), and support (10.85 ± 2.28). The average total SAS score was 41.70 ± 8.05, and that of EPDS was 6.82 ± 4.66.

**Fig. 1:**
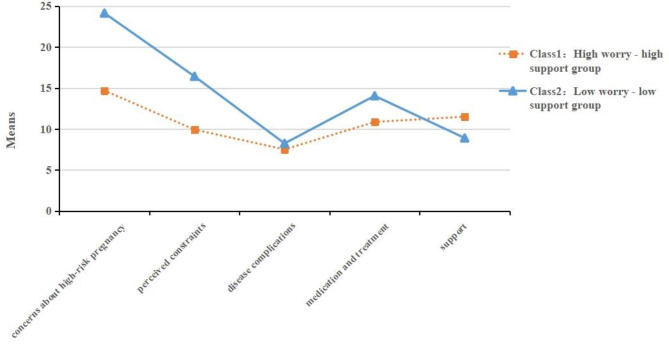
Two latent profiles of QOL in pregnant women with GDM: mean domain score by GDMQ latent profile

The model fit statistics for the five LPA models are outlined in Table 2. As the number of profiles increases, the AIC, BIC, and A BIC values gradually decrease. When only two profiles are retained, the entropy value reaches a maximum, and LMR and BLRT are statistically significant (p < 0.05). Thus, the two-profile solution was retained. The probability of each category of pregnant women belonging to each potential profile is 0.973 and 0.956, respectively, indicating that the models of the two potential profiles are reliable (Table 3). The potential section map is drawn according to the classification results, as shown in Fig. 1. There were 211 cases (75.6%) in group C1, and the scores of pregnant women in this category were significantly lower than those of the other group in the dimension of concerns about high-risk pregnancy, but their scores in the dimension of support were significantly higher than that of the other group. Therefore, group C1 was named the “high worry-high support” group. There were 68 cases (24.4%) in group C2, and the scores of pregnant women in this group were significantly higher than those in group C1 in concerns about high-risk pregnancy but significantly lower in the support dimension, so group C2 was named the “low worry-low support” group.

**Table 2:** Model fit indices for latent profile analysis

Profile	AIC	BIC	SABIC	Entropy	LMR *p*-value	BLRT *p*-value	Sample size by profile based on most likely membership
1	7013.205	7049.517	7017.808	1.000			1
**2**	**6686.177**	**6744.277**	**6693.542**	**0.891**	**0.000**	**0.000**	**75.6%/24.4%**
3	6640.473	6720.359	6650.599	0.731	0.147	0.000	20.8%/53.0%/26.2%
4	6605.765	6707.439	6618.653	0.815	0.849	0.000	2.9%/17.9%/20.8%/58.4%
5	6573.579	6697.040	6589.229	0.885	0.670	0.000	1.4%/15.1%/56.6%/5.4%/21.5%

Note: AIC: Akaike Information Criterion; BIC: Bayesian Information Criterion; LMR: Lo–Mendell–Rubin Adjusted Likelihood Ratio Test; BLRT: Bootstrapped Likelihood Ratio Test; the bold row was the chosen model

**Table 3:** Attribution probability matrix of each profile

Profile	Probabilities in profile	
	C1	C2
C1	0.973	0.027
C2	0.044	0.956

Note: C1 = high worry-high support group; C2 = low worry-low support group

Table 1 show that daily exercise time, disease duration, family history of diabetes, and anxiety have a predictive effect on the potential profile classification of QOL in pregnant women with GDM, and the differences are statistically significant (p < 0.05). In light of both the results shown in Table 1 and the clinical significance of depression [5], daily exercise duration, disease duration, family history of diabetes, anxiety, and depression were brought into the regression mixed model for analysis. The results show that daily exercise duration < 1 h (OR: 0.112; 95%CI: 0.880–1.439), daily exercise duration ≥ 1 h (OR: 0.049; 95%CI: 0.546–1.288), disease duration 2 weeks (OR: 0.058; 95% CI: 1.031–2.208), depression (OR:0.615; 95%CI: 1.184–1.819) were more likely to fall in the C1 “high worry-high support” group rather than the C2 “low worry-low support” group. Compared with the C1 “high worry-high support” group, pregnant women in the C2 “low worry-low support” group were more likely to have a family history of diabetes (OR: 7.68; 95%CI: 0.926–4.858), as seen in Table 4.

**Table 4:** Variables associated with profile membership

Predictor variable		C2 (low worry-low support group)		
		b (SE)	OR	95% CI
Duration of daily exercise (with “no exercise” as reference)	<1 h	−2.190 (0.941)*	0.112	0.880–1.439
	≥ 1 h	−3.023 (1.163)*	0.049	0.546–1.288
Disease duration (“disease duration 1 week” as reference)	Disease duration 2 weeks	−2.854 (0.999)**	0.058	1.031–2.208
	Disease duration 3 weeks	0.580 (0.876)	1.786	0.657–1.815
	Disease duration 4 weeks	0.077 (0.790)	1.080	0.666–1.944
Family history of diabetes (with “no family history of diabetes” as reference)		2.039 (0.766)**	7.680	0.926–4.858
Depression		−0.486 (0.133)***	0.615	1.184–1.819
Anxiety		0.030 (0.033)	1.031	0.824–2.328

Note: C1 “high worry-high support group” is the reference category

CI: Confidence interval OR: Odds ratio

## Discussion

The results of this study showed that the QOL of pregnant women with GDM had obvious classification characteristics, with two potential profiles: the C1 “high worry-high support” group (75.6%) and the C2 “low worry-low support” group (24.4%). All the evaluation indexes indicated that the model fit well, and that there was obvious heterogeneity in the QOL of pregnant women. The C1 “high worry-high support” group accounted for more than 75% of patients, indicating that the QOL of most pregnant women was at a low-to-medium level, which was consistent with the research results of Lee et al. [14]. This may be because GDM brings a variety of serious risks and adverse outcomes to pregnant women and their infants, so pregnant women not only have to bear the physical, psychological, economic, and social troubles of the disease, but also the worry about the safety and prognosis of the infant—seriously affecting their QOL.

The overall QOL level of the C1 “high worry-high support” group was relatively low, and only the score of the support dimension was high. This may be because pregnant women in this group had more pregnancy risk factors and GDM complications, which led to multiple symptoms bringing physical discomfort and negative emotions. At the same time, they also faced strict dietary restrictions and frequent blood sugar monitoring. These problems reduce their QOL but also make them receive more attention and support [13]. For such pregnant women, healthcare providers should provide practical and effective guidance and assistance to pregnant women according to their needs, and improve their ability to use social support through personalized interventions to improve their QOL. The C2 “low worry-low support” group had a higher overall QOL level and a low score only in the support dimension. This may be because pregnant women in this group had a higher sense of self-efficacy, strong self-management ability, and low demand for external support [28]. For such pregnant women, healthcare providers can encourage them to be actively involved in treatment decisions, learn to recognize changes in their condition, and be familiar with emergency treatment measures to further enhance their sense of self-worth [29].

This study showed that daily exercise duration < 1 h and daily exercise duration ≥ 1 h were more likely to belong to C1 “high worry-high support” group, and the probability of them entering C2 “low worry-low support” group gradually decreased with the increase of exercise time. However, Zhao et al. [30] showed that exercise can help improve blood sugar control, increase blood pressure and weight gain during pregnancy, and reduce insulin dosage in pregnant women with GDM, thereby improving their QOL. This is different from the results of this study, which may be because the exercise plan of pregnant women in this study was unreasonable and failed to exert the positive effect of exercise on improving QOL. Peters et al. [31] found that exercise is one of the most important measures to control blood sugar, but the effect of exercise on blood sugar control has great heterogeneity among various studies. This may be because the effect varies with exercise time, frequency, type, and intensity. Exercise guidelines for pregnancy issued by the American College of Obstetricians and Gynecologists (ACOG) recommend that pregnant women do 60–150 min of moderate-intensity aerobic exercise and resistance training per week, with an upper limit of exercise time of 30 min per day [32]. Therefore, a longer exercise time is not necessarily better, and, what is more, it is important to exercise scientifically within a reasonable time. In addition, although these pregnant women had high scores on the GDMQ support dimension, the support was mainly emotional and lacked professional exercise guidance. Therefore, healthcare providers must give GDM pregnant women without exercise contraindications more detailed and feasible individualized exercise guidance according to the guidelines to ensure safety and improve pregnant women’s QOL.

This study shows that pregnant women with a family history of diabetes are more likely to belong to the C2 “low worry-low support” group than those without a family history. This may be because pregnant women with a family history of diabetes pay more attention to diabetes-related knowledge and personal health than other pregnant women. They already have a certain knowledge of diabetes before the diagnosis of GDM, and they have a stronger ability to accept and cope with GDM—consistent with the findings of Guo et al. [33]. In addition, some pregnant women who have a family history of diabetes may have the experience of living with or caring for diabetic patients, which not only enables them to master a certain knowledge of diabetes but also enhances their self-efficacy and self-health management capabilities—consistent with the findings of Li et al. [34]. Kopec et al. [35] found that the QOL of pregnant women with GDM is closely related to disease knowledge. Iwanowicz-Palus et al. [28, 36] found that stronger disease acceptance and a higher level of self-efficacy can improve the QOL of pregnant women with GDM and reduce their need for external support. Therefore, healthcare providers should pay attention to disease health education for pregnant women, provide disease knowledge consultation and coping skills guidance, encourage pregnant women to self-monitor blood sugar, and carry out personalized interventions on pregnant women’s disease acceptance and self-efficacy. These can improve their disease acceptance and coping ability, enhance their confidence in self-health management, and improve QOL.

This study shows that pregnant women with more severe depression are more likely to belong to the C1 “high worry-high support” group, indicating that depression is a risk factor for poor QOL in pregnant women with GDM—consistent with the findings of Hong et al. [5]. The mental health of pregnant women with GDM is worse than that of normal pregnant women, and they are 2 to 4 times more likely to suffer from depression than pregnant women without GDM [37]. Depression will not only lead to hormone imbalance and elevated blood sugar in pregnant women but also increase the incidence of cesarean section and adverse maternal and child outcomes [5]. Xie et al. [13] found that depressed pregnant women with GDM have reduced utilization of social support and have serious concerns about the disease and treatment, which, in turn, promote the occurrence and development of depression, forming a vicious circle, where QOL drops further. Therefore, in addition to medication, healthcare providers need to pay attention to the negative mood of pregnant women. Healthcare providers can use cognitive behavioral therapy, mindfulness-based stress reduction therapy, and other psychosocial interventions to provide personalized psychological counseling for pregnant women. These can help pregnant women reduce psychological pressure, correctly understand GDM, and enhance treatment confidence; and thus prevent or alleviate prenatal depression, improve mental health during pregnancy, avoid adverse pregnancy outcomes, and promote maternal and child health.

The results of this study suggest that healthcare providers should provide personalized support to pregnant women with GDM. Previous studies have not only confirmed that this can improve the QOL score of pregnant women and improve the quality of life of pregnant women, but also has other benefits [38–41]. Research by Xue et al. [38] found that personalized support can also improve pregnant women’s medical compliance behavior. Studies by Zheng et al. [39] and Wang et al. [40] found that personalized support can also help pregnant women control their blood sugar within the normal range and improve their life satisfaction. Research by Li et al. [41] found that personalized support can also help pregnant women effectively reduce negative emotions and maintain a good mentality. Therefore, healthcare providers can combine the results of this study to assess the needs of different categories of pregnant women and provide corresponding support.

### Strengths and limitations

To our knowledge, this is the first study using a person-centered approach to analyze QOL in pregnant women with GDM. This study identified latent profile characteristics of QOL in pregnant women with GDM through LPA and explored the influencing factors of different profiles, which will help healthcare providers to develop more targeted QOL interventions for pregnant women. However, this study still has the following limitations. First, this study adopted convenience sampling, and the samples were only from pregnant women with GDM admitted to two hospitals in Guangdong Province, China. The sample can meet the requirements for statistical analysis, but whether the research results can represent women with GDM in other regions and countries still needs further verification. Second, this study found that the disease duration is an important factor affecting the quality of life of pregnant women with GDM, indicating that the quality of life of pregnant women with GDM is dynamically changing. However, this study is a cross-sectional study and cannot dynamically understand the relationship between quality of life and disease duration in pregnant women with GDM. In the future, a multi-center, large-sample longitudinal survey will be carried out to further verify the results of this study and analyze the dynamic interaction of QOL in different stages of pregnant women with GDM. In addition, we will also consider the impact of different treatment options (such as insulin treatment, dietary management, lifestyle management) on the quality of life scores of pregnant women with GDM.

## Conclusion

Our results showed heterogeneity in QOL in pregnant women with GDM. Based on LPA, this study divided the pregnant women into two profiles: a C1 “high worry-high support” group and a C2 “low worry-low support” group, and found that several factors such as daily exercise duration, disease duration, family history of diabetes, and depression degree can predict the potential profile of QOL in pregnant women. Healthcare providers should identify the profiles of QOL of pregnant women with GDM as early as possible, further assess their needs according to the characteristics and influencing factors of different profiles, and carry out personalized interventions based on needs to help them improve their QOL.

## Data Availability

The datasets generated and analysed during the current study are not publicly available due we may have further research related to the data but are available from the corresponding author on reasonable request.

## Abbreviations

GDM: Gestational diabetes mellitus
QOL: Quality of life
BMI: Body mass index
GDMQ: Quality of Life Scale for Women with Gestational Diabetes mellitus
LPA: Latent profile analysis
SAS: Self-rating Anxiety Scale
EPDS: Edinburgh Postpartum Depression Scale
AIC: Akaike information criterion
BIC: Bayesian information criterion
LMR: Lo–Mendell–Reuben-corrected likelihood ratio
BLRT: Bootstrap-based likelihood ratio
RMB: Renminbi
CI: Confidence interval
ACOG: American College of Obstetricians and Gynecologists
